# Robotic surgery: India is not ready yet

**DOI:** 10.4103/0970-1591.33717

**Published:** 2007

**Authors:** Vipul Patel

**Affiliations:** Director of Robotic Surgery, Associate Professor of Urology, Ohio State University, USA. E-mail: vipul.patel@osumc.edu

First of all I would like to congratulate the author on a well thought out argument. The article is quite provocative, chronicling the cold hard facts with a splash of wit thrown in for good measure. Well worthy of publication and discussion.

Is India ready for the robot? This is now becoming an age-old question. I must have been asked this question at least one thousand times by surgeons of Indian origin over the last three years. My response is quite simple: India does not have to prepare for robots, they are already there. Approximately five robots are already currently present in India and being used by various specialties. However, urology is really king when it comes to the robot. So the real question for Indian urologists is when will India take the next leap into the robotic revolution? As you see it's not a question of if, but rather when.

From an American perspective the future is clear. Robots are here to stay and have impacted the delivery of healthcare in a way that few technologies have in the history of surgery. Robotic technology has altered the fundamental foundations of surgery in the United States. There are now over 400 robots in the country and utilization rates are growing every year. In fact, it is estimated that in 2008 over 50% of all radical prostatectomies will be performed with robotic assistance. This represents a monumental milestone in surgery. The reason for this dramatic shift over essentially a five-year period is the rapid dissemination of the technology and technique. Robotic technology has provided some fundamental advantages to US surgeons. It has allowed those not laparoscopically trained to be able to offer their patients a minimally invasive alternative. For those who are laparoscopically trained it has given a platform for operating at a technically superior level.

Robotic technology has definitely received its share of marketing worldwide. However, marketing alone cannot account for its unprecedented growth and adoption rate. In the US, the dissemination of robotics has really been driven by patients and surgeons alike. Both with a common goal: less invasive care with superior outcomes. The addition of the evidence-based approach to evaluation of the procedure has shown that the outcomes are definitely no worse than open or laparoscopic surgery but in many cases quite superior. Being trained as first an open surgeon and then as a standard laparoscopic surgeon, this has given me a unique insight into the advantages of the technology. With experience, one can perform the procedure with improved efficiency and optimal outcomes. My own experience is now over 1700 robotic prostatectomies and 100 pyeloplasties. Robotic technology seems uniquely suited for reconstructive urology, especially in the tight confines of the pelvis. Our experience has allowed us to enter a new generation of radical prostatectomy. The average operative time is now under 1h and 15 min, blood loss < 100 cc and patients are discharged home the day following surgery with little to no need for narcotic medication. They return at Day five for catheter removal. In the majority total continence is expected at six to eight weeks and potency by three to six months. We have come a long way but the fun is just beginning.

In India the assimilation of robotic technology has already occurred but has not expanded or entered the mainstream. There remains a lack of access to the technology and a deficit in educational opportunities. The reason undoubtedly is not for a lack of utility of the instrumentation or the lack of benefit to the patient. The reason has primarily been cost. Robotic technology is expensive and is showing no signs of becoming cheaper. There is one robot (daVinci, Intuitive Surgical. Sunnyvale, CA USA) in the world with the utility that we desire at the present time and therefore the technology is at a premium price. The price tag is unlikely to come down in the near future, so what should we do? Shun the technology until the next generation, lobby the company for specialized pricing or make a stride into the new era of surgery? The choice is yours.

Robotic technology is in India and will without question grow in the near future. I do not advocate the random, rapid, uneducated expansion of robotics in India. The technology requires education and training for safe implementation. It also requires economic backbone; the cost of this advanced technology must be taken on by either the hospital, surgeon or the patient. The best approach is likely an evidence-based evaluation of the technology at centers with expertise in laparoscopic procedures, at institutions where there are experts in the field of prostatectomy and where the volume is substantial enough to allow frequent enough utilization to keep the surgeon and the team experienced.

India should not ignore the robotic revolution that has swept through the US and is now going through Europe, Asia and Latin America. After all India claims that it will soon be a “superpower”. With this tag comes much expectation and responsibility. The population must have access to all types of medical technology and the surgeons must be trained in the latest practices and techniques. If India does not at least fairly evaluate the technology, give opportunity for training of its surgeons or provide it as an alternative for patient care then it faces some grim possibilities. It is evident from spending time in India that there is great interest, excitement and cautious optimism. The young surgeons in India have been intrigued by the technology and have developed a thirst for evaluating it on their home soil. Currently, this is not possible for them. Therefore, in the US we have seen a huge influx of exceptional young trained Indian urologists, seeking to be trained in robotic surgery. It has been our distinct pleasure and honor to welcome these young trailblazers. We have embraced their enthusiasm and have created positions so that they can fulfill their dreams. This is a duty that we have fulfilled with great pleasure. What India stands to lose is its next generation of the brightest minds and spirit for innovation, reverse outsourcing so to speak. This year alone we have three of India's brightest potential stars expanding their horizons at our institution. However, the question is not when will they return, but will they want to?

So the challenge for all Indian urologists is to educate themselves first about the values of the next generation of technology, examine the alternatives and then selectively apply it in an evidence-based manner. This approach provides the most likely opportunity for patient safety and surgeon success. I have found this technology to be effective, worthy of the investment and it has undoubtedly positively affected the care of my patients. India, our budding “Superpower” welcome to the robotic revolution. We have been waiting for you.

Warmest regards.

